# Supplementary material for: Oxytocin neurons signal state-dependent transitions from rest to thermogenesis and behavioral arousal
Source: eLife. 2026 Oct 30;14:RP108212. doi: 10.7554/eLife.108212 (PMC13722414; doi:10.7554/eLife.108212)
Supplement: Peer review history

# Editor report: eLife Assessment

**DOI:** 10.7554/eLife.108212.4.sa0

**Authors:** Moriel Zelikowsky

**Affiliations:** University of Utah United States

**Keywords:** Important, Solid

This study presents an **important** finding regarding the role of oxytocin neurons in thermogenesis and behavioral thermoregulation. The use of numerous converging methods, including behavior, fiber photometry, optogenetics, thermal recordings, metabolic analyses, and more, produces a multi-dimensional dataset delivering findings that provide **solid** support for the conclusions. The conclusions could be further strengthen by more extensive analyses of behavior and determining whether it is the release of oxytocin (rather than co-release of glutamate) from the PVN that is critical for the transition between behavioral states, nevertheless, the manuscript had many strengths, the findings are novel, and this work opens new doors for understanding the role of the PVT in thermoregulation. This work will be of strong interest to the thermoregulation, social behavior, and oxytocin signaling communities.

# Referee report: Reviewer #1 (Public review)

**DOI:** 10.7554/eLife.108212.4.sa1

Summary:

The authors identify and investigate a specific population of PVNOT neurons (oxytocin neurons of the paraventricular hypothalamus) that seem to be involved in both behavioral and autonomic thermoregulation. These cells are activated by social thermoregulatory behaviors, but can influence thermoregulation in both social and social contexts, specifically during transitions and when mice are at low core body temperature (Tb).

Comments on revised version.

The authors have addressed my concerns with clear and reasonable explanations and altered the text accordingly. This has improved the paper, but it still feels in some parts like a patchwork of nice work and discoveries stitched together. Further changes to format, analysis, and some experimental work could hugely improve the manuscript. I see that will surely come from future work, and this is the authors' choice.

Regarding the lack of behavioral analysis, I think it's fair for them to keep it for future studies.

I am happy to see they take and expand the opto inhibition suggestion. Again, that experiment would be nice for this paper, but not crucial.

Regarding discussing Raam et al 2026. It is good that they detail the practical decision of using females. What I meant was that, given that both papers study calcium dynamics around the time when mice engage in social thermoregulatory behaviour, they could have speculated on potential dmPFC-PVN functional connectivity, for example. Or the fact that Raam found that females showed fewer huddling behaviour than males at 5{degree sign}C (however, Vandendoren tested 15{degree sign}C, not 5{degree sign}C). Discussion of these features would be welcome, but maybe all of the current scope.

Overall, this is a very strong paper.

# Referee report: Reviewer #2 (Public review)

**DOI:** 10.7554/eLife.108212.4.sa2

This is a very interesting study from Vandendoren and colleagues examining the role of PVN oxytocin neurons during thermoregulatory behaviors, in particular during thermoregulatory huddling. The findings are important and have implications for the thermoregulation field as well as the social/naturalistic behavior field. The findings are compelling and use a combination of state-of-the-art tools (photometry, optogenetics, automated behavior tracking, thermal imaging, and core body temperature measurement), often in combination with each other, to produce a rigorous and high-dimensional dataset.

Comments on revised version.

I appreciate the effort the authors have put into addressing all of my questions, and I have no remaining concerns.

# Referee report: Reviewer #3 (Public review)

**DOI:** 10.7554/eLife.108212.4.sa3

Summary:

This study investigates how the activity of hypothalamic paraventricular oxytocin (PVNOT) neurons relates to physiological states in female mice, with a particular focus on behavioral states and thermogenic sympathetic activity. To address this question, the authors combined automated video-based behavioral classification with calcium imaging of PVNOT neuron activity. Sympathetic thermogenesis was inferred from surface temperature changes measured by infrared thermography, and the authors have made their custom analysis scripts available. The authors report that strong, pulsatile activation of PVNOT neurons was "occasionally" observed immediately before transitions from resting to active states. This observation suggests that PVNOT neuronal activity may facilitate the transition from rest to activity. This phenomenon was observed in both pair-housed and individually housed animals. Taken together, these findings raise the possibility that the oxytocinergic system contributes to naturalistic behavior transitions even in the absence of social interactions. However, concerns regarding the selectivity of GCaMP expression in oxytocin-expressing neurons call into question the validity of the recorded PVNOT neuronal activity. The revised manuscript improves the presentation and interpretation of the data. Nevertheless, because the authors have not provided additional experiments or analyses addressing the major methodological concerns, the evidence supporting the central conclusions remains essentially unchanged.

Strengths:

The oxytocinergic neural system is believed to subserve a wide range of physiological functions. Elucidating these roles requires monitoring PVNOT neuronal activity under diverse behavioral contexts, as well as manipulating this activity to establish causal relationships. In this study, the authors present a technically sound experimental framework that integrates behavioral tracking in both individually and group-housed mice with the monitoring and manipulation of PVNOT neuron activity. This setup represents a valuable methodological resource for researchers investigating the physiological functions of oxytocin.

Weaknesses:

(1) Immunohistochemical validation of selective GCaMP expression in oxytocin-expressing neurons showed that only 24-51% of GCaMP-positive neurons expressed oxytocin. As an alternative approach, the authors argue that the similarity between calcium dynamics recorded in virgin and lactating animals supports the identity of the recorded neurons as oxytocin neurons. While this physiological comparison is interesting, it does not constitute direct evidence for cell-type specificity of GCaMP expression. The revised manuscript now acknowledges that in situ hybridization targeting oxytocin mRNA would provide a more reliable validation, but such validation has not been performed. Therefore, uncertainty regarding the identity of the recorded neurons remains, limiting confidence in the interpretation of the calcium imaging data.

(2) Although the authors' interpretation is generally consistent with the data presented, their main conclusions rely heavily on observational findings. Moreover, optogenetic stimulation of PVNOT neurons failed to robustly recapitulate behavioral state transitions (Figs. 6D and S5B). Further interventional experiments remain necessary to rigorously test the authors' interpretation and establish a causal relationship between PVNOT activity and rest-to-active transitions. In particular, loss-of-function approaches targeting the PVNOT system, such as OXTR antagonism, inhibitory optogenetics, or cell-type-specific ablation, remain essential to determine whether perturbation of this system alters behavioral state transitions. Although the authors expanded the Discussion to acknowledge this limitation, the revised manuscript provides no additional experimental evidence addressing it.

Comments on revised version.

I appreciate the authors' efforts to clarify the manuscript and to discuss the limitations more explicitly. Nevertheless, because my major concerns have been addressed primarily through revised interpretation rather than new evidence, my overall assessment of the scientific support for the principal conclusions remains unchanged.

# Author comment: Author response

**DOI:** 10.7554/eLife.108212.4.sa4

**Authors:** Morgane Vandendoren, Jason G Landen, Joseph F Rogers, Samantha Killmer, Baizar Alamiri, Celeste Pohlman, Glenn Tattersall, Nicole L Bedford, Adam Nelson

**Affiliations:** Creighton University Omaha United States; University of Wyoming Laramie United States; University of Wyoming Wyoming United States; University of Wyoming Laramie United States; University of Wyoming Laramie United States; University of Wyoming Laramie United States; Brock University Ontario Canada; University of Wyoming Laramie United States; University of Wyoming Wyoming United States

The following is the authors’ response to the previous reviews.

**Public Reviews:**

**Reviewer #1 (Public review):**

Comments on revised version.

As discussed before, the authors employ a wide range of techniques (FOS IHC, FP for fine scale PVN OXT population dynamics, behavioural analysis, core and surface temperature tracking, physiological recordings to assess AAV specificity, optogenetic activation of PVN OXT neurons, and projection tracing) to address a clear question. The outcomes of these techniques seem to drive the same conclusion that PVN OXT neurons signal transitions from rest to arousal (behavioural and thermogenic) in a state-dependent manner:

- FOS data identifies PVN OXT population activity following behavioural onset

- Ca activity in these cells peaks at behavioural and thermogenic state transitions

- Rump temperature and BAT activity increase at state transition points

- Optogenetic stimulation of these cells recapitulates the thermogenic effects seen during physiological state transitions (in low body temperature animals) with a trending increase in physical activity

Despite the inconclusive IHC results when validating the specificity of their AAV, the virgin female/ lactation experiment is convincing that they are specifically targeting PVN OXT neurons. The rationale for this experiment is clearer in the revised manuscript.

Generally, in terms of the revised manuscript, the authors give strong responses to reviewer comments, either incorporating feedback, or giving clear explanations for the choices they made in the original manuscript. The revised manuscript is clearer about the question the authors aim to address, the reasons for their choice of experiments, and the limitations of the techniques used.

We thank the reviewer for the close attention to the manuscript, the response to reviewers, and the revision, all of which have improved the manuscript.

Criticisms:

I appreciate and agree with the authors' point that this manuscript is more fundamental than simply social basis oxytocin neuron function. This is point is well made by their data, and in the revised text. However, I still believe more behavioural analysis would be welcome to any reader.

They partly justify the lack of behavioural analysis in Figure 6 with the problem of "animal merging" on the SGBS images. However, in Figure 6C, they confirm that, in solo conditions, the SGBS readings are consistent with core body temperature readings. So why not stick to core body temperature, opto stimulate and analyse the social behaviour with DLC (with normal video recordings)?

This is a good suggestion. Because we find that quiescent huddling (paired) bouts were associated with stronger body temperature regulation compared to solo quiescence and other behavioral states, and because PVNOT peak probability and frequency were higher in the paired compared to solo context, these experiments are warranted. We made the following edits to the discussion:

“Future experiments should attempt to disentangle the effects of PVNOT light stimulation on social vs. non-social aspects of these behavioral state transitions; of particular interest would be to examine how light stimulation affects the duration and thermoregulatory control of social huddling.”

The lactation validation still seems out of place in manuscript order. It is a very valuable validation, but it feels more like supplementary data for Figure 1. I feel the authors wanted it as a main figure because of how much work it must have been. In that case, it still makes more sense to include it in Figure 1.

The purpose of the lactation experiment arose from the inadequacy of using histology to test whether AAV-transfected cells were oxytocin-immunoreactive. Because we observed intense oxytocin immunoreactivity in the fibres lining the ventricle, and less reactivity in the cell bodies than what we would have predicted from the Oxytocin-Cre-dependent AAV, we turned to the known physiological relationship between oxytocin-positive neurons and lactation. As such, this study is not associated with Figure 1, which demonstrates our initial, coarse-grained findings relating FOS activity in the PVN and in oxytocin-positive neurons during social thermoregulation.

To your point, it typically does make sense to have the cellular validation “up front” as supporting or background information that enables the downstream experiments. However, what gives this data credibility as a standalone figure is the novel finding that PVNOT neurons display burst-like patterns of activity outside the context of lactation. Previous discussions with experts in the field, along with a review of the literature, unexpectedly led us to the observation that the burst-like patterns we observed during the transition from rest to wake and thermogenesis in virgin females represents a new aspect of oxytocin neuron physiology. Because we wanted to directly compare the new virgin female activity pattern (i.e., Figure 2) with the known lactation activity pattern, we decided it made the most sense to combine the validation aspect with the novel aspect into a standalone figure.

Though their lactation experiment validates that they are targeting PVN OXT neurons, their optogenetic stimulation protocol may not be specifically inducing OXT release from these cells. PVN OXT neurons co-release glutamate but can also release glutamate independently of OXT following lower frequency tonic stimulation. OXT release from PVN neurons requires pulsatile stimulation at a higher frequency (Leithead et al., 2021; Piñol et al., 2014; Lincoln & Wakerley, 1975). In this paper, the authors use a low stimulation frequency (10Hz) and continuous pulse train (20s) to optogenetically manipulate the target PVN population which may bias the cells towards glutamate release over OXT. Therefore, though they find evidence that PVN OXT neurons are involved in driving the transition between states in their other experiments, their optogenetic stimulation may not necessarily involve OXT release/signalling. It may be valuable to separate this out to identify the signalling molecule underlying this behavioural/ thermogenic transition. This could be done by using an opto protocol that recapitulates physiological OXT release.

The authors do however mention that isolating the specific contribution of OXT signalling compared to other co-transmitted molecules was not the aim of this study, so this is not an essential question for this manuscript.

Thank you for this thoughtful point. We agree our optogenetic stimulation experiment should be interpreted as activation of PVNOT neurons rather than as selective evidence for oxytocin release or oxytocin signaling. PVNOT neurons can co-release glutamate (an idea we had also briefly touched upon in the Limitations and caveats section), and the stimulation pattern/frequency may influence the relative engagement of fast glutamatergic transmission versus peptide release. We agree the lactation literature, including Lincoln et al., highlights the importance of high-frequency pulsatile activity for oxytocin release, and that Piñol et al. provide evidence that PVNOT-linked glutamatergic transmission can interact with oxytocin-receptor-dependent modulation of downstream synapses–so thanks for pointing these out.

We made revisions to support our protocol and now acknowledge this important aspect of the neuronal physiology. In Results, we now explain why we selected 10Hz: this frequency was grounded in the study by Fukushima et al. (2022), where 10Hz stimulation of PVNOT terminals in the rMR elicit thermogenic responses and 10Hz stimulation of PVNOT somata produce thermogenesis that’s dependent on oxytocin receptors in rMR.

In the Limitations section, we now cite these three references to include broader context around stimulation frequency and differential release. We emphasize that our optogenetic data demonstrate sufficiency of PVNOT neuron activation, but do not establish whether the downstream thermogenic and behavioral effects are mediated by oxytocin, glutamate, or both. We note that resolving this issue will require future experiments using stimulation-pattern comparisons together with receptor-targeted pharmacology or genetic loss-of-function approaches.

References

Leithead, A. B., Tasker, J. G., & Harony-Nicolas, H. (2021). The interplay between glutamatergic circuits and oxytocin neurons in the hypothalamus and its relevance to neurodevelopmental disorders. Journal of neuroendocrinology, 33(12), e13061. https://doi.org/10.1111/jne.13061

Lincoln, D. W., & Wakerley, J. B. (1975). Factors governing the periodic activation of supraoptic and paraventricular neurosecretory cells during suckling in the rat. The Journal of physiology, 250(2), 443-461. https://doi.org/10.1113/jphysiol.1975.sp011064

Piñol, R. A., Jameson, H., Popratiloff, A., Lee, N. H., & Mendelowitz, D. (2014). Visualization of oxytocin release that mediates paired pulse facilitation in hypothalamic pathways to brainstem autonomic neurons. PloS one, 9(11), e112138. https://doi.org/10.1371/journal.pone.0112138

A loss of function experiment to test for sufficiency would be a nice addition to further confirm their claims, but the authors mention that there were technical limitations to their attempts at inhibiting PVN OXT neurons. I appreciate the authors declaring that the DREADDs attempt suffered from unfortunate confounds. But for optogenetic attempts, I don't think they need a closed-loop system to get some useful results. They still can shine the light at "random" moments (that will correspond to random body temperatures) and then separate the data per body temperature.

We thank the reviewer for this constructive suggestion. Such an experiment would strengthen our claims and complement the optogenetic activation (Fig. 6). Reviewer 3 brought up a similar concern.

Building directly on the reviewer’s proposal, we now describe a loss-of-function experiment as an important next step. Optogenetic inhibition of PVNOT neurons can be delivered at pseudo-random times across light and rest phase. Because animals spend extended periods at rest during this phase, a substantial fraction will fall within established rest bouts, which can then be analyzed and stratified by body temperature, as the reviewer notes. The prediction is that silencing PVNOT neurons during rest should prolong the average duration of rest bouts and delay the onset of activity and thermogenesis, relative to matched unstimulated bouts.This provides a direct test of whether PVNOT activity is necessary for the transition from rest to activity. We have revised the Limitations and caveats section to describe this experiment.

“Third, although we show that PVNOT neurons are sufficient to drive thermogenic and behavioral transitions (Fig. 6), we did not perform acute loss-of-function experiments. Such experiments are warranted because decreases in baseline PVNOT calcium activity were associated with transitions toward the onset of quiescence (Fig. 3I-L), suggesting this system may bidirectionally regulate thermo-behavioural state. A tractable next step would be to optogenetically inhibit PVNOT neurons during established rest bouts, delivered at pseudo-random times across the light and rest phase and analyzed post hoc by behavioral state and body temperature; we predict that silencing during rest would prolong the average duration of rest bouts and delay the onset of activity and thermogenesis. Pairing the inhibition with selective oxytocin antagonist (such as L-368,899), would further test whether the thermogenic and autonomic components of these transitions are oxytocin receptor dependent rather than driven by glutamate released by the same neurons.”

Lastly, the mention of Raam et al. 2026 is insufficient. The authors just mention it regarding the potential differences with males, to be explored in future experiments. Even if not using males in the current study doesn't affect the stated conclusions, the fact that they chose females because "their thermo-behavioural states were readily discernible" is a considerable bias. Testing males in this very study might be out of scope, but more discussion is warranted.

We thank the reviewer for this point. We agree that our decision to study females deserves fuller treatment, and we have expanded the Limitations and caveats section accordingly.

We want to be clear about the rationale, because it was methodological rather than an assumption of sex specificity. Our previous study on behavioral thermoregulation in mice (Landen et al., 2024) showed that, during the light/rest phase, females–but not males–display clearly rhythmic episodes of rest and activity that align with transitions between thermoregulatory states, and are therefore well suited to the analyses that form the core of this study. This choice does constrain the generality of our findings to females, but it does not affect the validity of the conclusions we draw, all of which concern PVNOT neurons in females.

At the same time, we agree that whether these mechanisms extend to males is a substantive open question and we now say so explicitly. A direct comparison in males, while beyond the scope of the present study, is an important next step, and the recently defined neural basis of collective thermoregulatory huddling (Raam et al. 2026) offers a useful framework for that work. We have modified the Discussion/Limitations and caveats as follows:

“We focused on females for a practical reason: during the light and rest phase, females show clear, rhythmic bouts of rest and activity, which makes transitions between thermoregulatory states readily discernible and well suited to the analyses around each state transition used here (Landen et al., 2024). This choice constrains the generality of our conclusions, which pertain specifically to females. Because oxytocin signaling can differ between sexes (https://doi.org/10.1016/j.yfrne.2015.04.003), and because the neural control of thermoregulatory behavior may not be identical in males, whether the PVNOT dynamics we describe operate similarly in males remains an open question. Testing males directly was beyond the scope of the present study, but it is an important next step, particularly as the neural basis of collective thermoregulatory huddling has recently begun to be defined (Raam et al. 2026).”

**Reviewer #2 (Public review):**

Summary:

This is a very interesting study from Vandendoren and colleagues examining the role of PVN oxytocin neurons during thermoregulatory behaviors, in particular during thermoregulatory huddling. The findings are important and have implications for the thermoregulation field as well as the social/naturalistic behavior field. The findings are compelling and use a combination of state-of-the-art tools (photometry, optogenetics, automated behavior tracking, thermal imaging, and core body temperature measurement), often in combination with each other, to produce a rigorous and high-dimensional dataset.

Comments on revised version.

I appreciate the effort the authors have put into addressing all of my questions, and I have no remaining concerns.

Thanks for the comments; they have greatly improved the manuscript.

**Reviewer #3 (Public review):**

Summary:

This study investigates how the activity of hypothalamic paraventricular oxytocin (PVNOT) neurons relates to physiological states in female mice, with a particular focus on behavioral states and thermogenic sympathetic activity. To address this question, the authors combined automated video-based behavioral classification with calcium imaging of PVNOT neuron activity. Sympathetic thermogenesis was inferred from surface temperature changes measured by infrared thermography, and the authors have made their custom analysis scripts available. The authors report that strong, pulsatile activation of PVNOT neurons was "occasionally" observed immediately before transitions from resting to active states. This observation suggests that PVNOT neuronal activity may facilitate the transition from rest to activity. This phenomenon was observed in both pair-housed and individually housed animals. Taken together, these findings raise the possibility that the oxytocinergic system contributes to naturalistic behavior transitions even in the absence of social interactions. However, concerns regarding the selectivity of GCaMP expression in oxytocin-expressing neurons call into question the validity of the recorded PVNOT neuronal activity.

Strengths:

The oxytocinergic neural system is believed to subserve a wide range of physiological functions. Elucidating these roles requires monitoring PVNOT neuronal activity under diverse behavioral contexts, as well as manipulating this activity to establish causal relationships. In this study, the authors present a technically sound experimental framework that integrates behavioral tracking in both individually and group-housed mice with the monitoring and manipulation of PVNOT neuron activity. This setup represents a valuable methodological resource for researchers investigating the physiological functions of oxytocin.

Thanks for the comments. We are encouraged to hear this framework will open new doors in understanding how the oxytocin system regulates behavior and energy homeostasis.

Weaknesses:

(1) Immunohistochemical validation of selective GCaMP expression in oxytocin-expressing neurons showed that only 24-51% of GCaMP-positive neurons expressed oxytocin. As an alternative approach, the authors demonstrate that GCaMP-expressing PVN neurons in virgin females exhibit calcium peaks during rest-wake transitions with kinetics similar to those observed in PVNOT neurons during early lactation. However, this comparison is based solely on population-level peak profiles and does not provide direct evidence for cell-type specificity of GCaMP expression in oxytocin neurons. This limitation substantially undermines the validity of the optical calcium imaging data. In situ hybridization targeting oxytocin mRNA, rather than immunohistochemistry, may provide a more reliable assessment of expression specificity.

We view our data as showing strong evidence that the recorded neurons include, but may not be limited to, PVNOT neurons for the following two reasons: (1) as the reviewer notes, our longitudinal experiment shows conservation in the physiological and biophysical profile of these neurons in females that went from virgins to parturition and lactation, and (2) as described in Discussion/PVNOT neurons in context of arousal and peptidergic PVN cell-types, non-OT cell-types in the PVN do not show this pulsatile busting profile.

In the “Discussion/Thermal tracking and validation of PVNOT recording specificity” section we had stated “We note that the animals were perfused at ~ZT4–8, before we were aware that somatic OT immunoreactivity in PVN neurons reaches a daily low during the early light phase [56]”. We now add to this the idea, suggested by the reviewer, that “In situ hybridization targeting oxytocin mRNA, rather than immunohistochemistry, may provide a more reliable assessment of expression specificity.”

(2) Although the authors' interpretation is generally consistent with the data presented, their main conclusions rely heavily on observational findings. Moreover, optogenetic stimulation of PVNOT neurons failed to robustly recapitulate behavioral state transitions (Figs. 6D and S5B). Further interventional experiments will be necessary to more rigorously test the authors' interpretation and to establish mechanistic insight into the causal relationship between PVNOT activity and rest-to-active transitions. In particular, loss-of-function approaches targeting the PVNOT system, such as OXTR antagonism, inhibitory DREADDs, or cell-type-specific ablation, will be essential to determine whether perturbation of this system alters behavioral state transitions These points should be addressed in future studies.

Reviewer 1 brought up a similar concern. We have added to the Discussion/Limitations and caveats to address this.

“Third, although we show that PVNOT neurons are sufficient to drive thermogenic and behavioral transitions (Fig. 6), we did not perform acute loss-of-function experiments. Such experiments are warranted because decreases in baseline PVNOT calcium activity were associated with transitions toward the onset of quiescence (Fig. 3I-L), suggesting this system may bidirectionally regulate thermo-behavioural state. A tractable next step would be to optogenetically inhibit PVNOT neurons during established rest bouts, delivered at pseudo-random times across the light and rest phase and analyzed post hoc by behavioral state and body temperature; we predict that silencing during rest would prolong the average duration of rest bouts and delay the onset of activity and thermogenesis. Pairing the inhibition with selective oxytocin antagonist (such as L-368,899), would further test whether the thermogenic and autonomic components of these transitions are oxytocin receptor dependent rather than driven by glutamate released by the same neurons.”

Note: as described in the previous response to reviewers, we have tried inhibitory DREADDs in this system and have concluded that it is of little value because delivering DREADD ligand requires handing the animals for an IP injection—a procedure that disrupts sleep/rest and induces stress hyperthermia.

**Recommendations for the authors:**

**Reviewer #1 (Recommendations for the authors):**

The authors have answered our criticisms and can proceed as they chose. This is an important paper, and it is the author's choice whether to develop their research here or in a subsequent paper.

Thank you.

**Reviewer #2 (Recommendations for the authors):**

I thank the authors for citing my pre-print, as suggested by Reviewer 1. The paper has now been published and the authors may like to cite the published version (doi.org/10.1038/s41593-026-02224-0).

Thank you.

**Reviewer #3 (Recommendations for the authors):**

(1) The authors now interpret their results as indicating that PVNOT activity biases the system toward state transition (from rest to active), rather than acting as a deterministic trigger. This interpretation is reasonable. However, the wording "PVNOT peaks (or neurons) predict transitions to behavioral arousal and thermogenesis" may be misleading. If arousal and thermogenesis occur in more than 80% of cases following PVNOT peaks, then such peaks could reasonably be described as "being predicted". Otherwise, the terminology should be revised for clarity.

We thank the reviewer for raising this question, which touches on a substantive issue in how predictive relationships are characterized. We agree that "predicts" can misleadingly imply a high positive predictive value: i.e., that a large fraction of peaks are followed by transitions.

This is not the claim we intend, nor is it the appropriate statistical criterion. A variable is predictive when it shifts the conditional probability (or, here, the conditional distribution) of the outcome relative to its base rate — the criterion underlying likelihood ratios, relative risk, and signal-detection measures — rather than when it exceeds an absolute occurrence threshold such as 80%. By this standard, a peak can be informative even if transitions do not follow the majority of peaks, provided transitions are substantially more likely (or thermogenically warmer) when a peak precedes them than when one does not.

Our data support precisely this. The logistic regression shows peaks are much more probable immediately before rest offset than at other transitions or at baseline, and our new analysis shows that transitions preceded by peaks carry significantly larger post-offset Tb increases than those without. We are not claiming peaks act as a deterministic trigger, and we agree with the reviewer that they are not present before every transition.

To keep our language aligned with these results, we have revised the wording to avoid "predict" where it could imply high hit-rate determinism, replacing it with comparative phrasing. Accordingly, we have revised the terminology throughout the manuscript: where a claim concerns timing, we now state that peaks “precede” transitions. We have removed “predict”/”predictive” from the section heading, figure legend, introduction and results as follows.

“Then, we discovered that PVNOT calcium dynamics during huddling were associated with increased likelihood of transitions to body warming and arousal.”

“PVNOT neuronal activity precedes transitions towards thermogenesis and behavioral arousal in social and non-social contexts.”

Fig. 3 legend title: “PVNOT peaks are associated with increased likelihood of thermogenic rest-to-active transitions.”

“Thus, PVNOT peaks are at least five-fold more likely to occur near the offset of quiescence/quiescent compared to onset, and signal an increase in physical activity—a correlate of behavioral arousal 53 and a means of increasing metabolic rate and Tb [26]”

“Thus, for nesting and active huddling, PVNOT peaks are two- to three- fold more likely to occur at bout onset than offset.” Dropping flagged word here lol.

“Together these results suggest that elevated PVNOT activity dynamics precede the offset of two rest states (quiescence and quiescent huddling) by approximately 100 seconds, and the onset of two post-quiescence active states (nesting and active huddling) by around 20 seconds, in solo and paired mice respectively.”

“Moreover, PVNOT peaks aligned with the low point of a U-shaped body temperature profile: on average, Tb decreased before, and increased after, the time of the calcium peak in both solo and paired conditions (Fig. 3O,R). Together, these results suggest that PVN^OT^ peaks occur during a low Tb trough and mark a subsequent rise in Tb.”

(2) Regarding the 400-sec latency of BAT surface temperature increases following optogenetic stimulation, the authors now attribute this delay to slow peptidergic transmission. However, the authors should consider prior findings showing that BAT temperature increased immediately following optogenetic stimulation of PVN→rMR oxytocin neurons in anesthetized rats (Fukushima et al., 2022).

My hunch is that doing this in anesthetized rats gives a stronger signal to noise… not sure if I can back that up though.

At the least we can add a sentence that says “rMR oxytocin neurons immediately increases BAT temperature, while infusion of OXT or NMDA in the rMR results in BAT temperature increases after approximately one minute…” (see Fig. 3,4,5).

We thank the reviewer for redirecting us to Fukushima et al. (2022). We note, however, that in that study the fast-responding variable was BAT sympathetic nerve activity, whereas the BAT temperature itself rose over several minutes following both optogenetic stimulation (their Fig. 4F, quantified at 5 and 10 minutes) and focal rMR infusion of oxytocin or NDMA (their Fig. 5, multiminute traces). This thermal timescale is comparable to the one we observe.

The remaining difference could reflect methodological differences: we stimulated PVNOT somata rather than rMR terminals, measured interscapular surface rather than BAT temperature directly, and recorded in awake, freely behaving animals (rather than anesthetized animals) in which competing thermoeffector and behavioral processes are active. Consistent with a methodological basis for the delay, focal infusion of oxytocin or NMDA into the rMR in that study increased BAT temperature over roughly a minute (Fukushima et al., 2022). Slow, diffuse peptidergic neuromodulation may further contribute, oxytocin is released from large dense-core vesicles and can act over extended time scales (Ludwig and Leng, 2006; Parmaksiz and Kim, 2025; Qian et al., 2023), although our data cannot isolate this mechanism from the factors above or from fast glutamatergic co-transmission that likely accompanies PVNOT activation (Hrabovszky and Liposits, 2008).

(3) In the previous review, clarification was requested regarding the rationale and histological basis for intravenous FluoroGold injection. While the authors have now added methodological details, they should also incorporate the following explanatory text (previously provided in their rebuttal) into the manuscript for readers unfamiliar with PVN histological analyses:

"Intravenous injection of FluoroGold (FG) was used to histologically differentiate between magnocellular and parvicellular oxytocin neurons in the PVN. Because the posterior pituitary is located outside the blood-brain barrier, i.v. FG is selectively taken up by terminals of magnocellular neurons and retrogradely transported to their cell bodies. This allows us to infer the neuroanatomical identity (magno- vs. parvicellular) of the PVNOT neurons of interest."

We thank the reviewer for this suggestion. We have added the explanatory text to the results subsection, “PVN^OT^ cellular projections to the rMR”. The text now reads: “rMR cell types in mice, we used FluoroGold FG to disambiguate magno- vs. parvocellular PVN^OT^ projections [67] (Fig. S6A-C). Because the posterior pituitary is located outside the blood-brain barrier, intravenous FG is selectively taken up by terminals of magnocellular neurons and retrogradely transported to their cell bodies. This allows us to infer the neuroanatomical identity (magno- vs. parvicellular) of the PVN^OT^ neurons of interest.”
